# SIV/SARS-CoV-2 coinfection in rhesus macaques impacts viral shedding, host immunity, the microbiome, and viral evolution

**DOI:** 10.3389/fimmu.2025.1587688

**Published:** 2025-05-20

**Authors:** Megan N. Fredericks, Zohar Kolodner, Adam Waalkes, Kaitlin Sawatzki, Linhui Hao, Dustin R. Long, Kelsi Penewit, Cecily C. Midkiff, Carter J. McCormick, Semira Beraki, Paul T. Edlefsen, Jeana Barrow, Alexander L. Greninger, Michael Gale, Robert V. Blair, Stephen J. Salipante, Deborah H. Fuller, Megan A. O’Connor

**Affiliations:** ^1^ Department of Microbiology, University of Washington, Seattle, WA, United States; ^2^ Washington National Primate Research Center, Seattle, WA, United States; ^3^ Department of Laboratory Medicine and Pathology, University of Washington, Seattle, WA, United States; ^4^ Department of Immunology, University of Washington, Seattle, WA, United States; ^5^ Division of Critical Care Medicine, Department of Anesthesiology and Pain Medicine, University of Washington School of Medicine, Seattle, WA, United States; ^6^ Tulane National Primate Research Center, Covington, LA, United States; ^7^ Fred Hutchinson Cancer Research Center, Seattle, WA, United States; ^8^ Department of Microbiology and Immunology, University of Minnesota Twin Cities, Minneapolis, MN, United States

**Keywords:** HIV, immunocompromised, COVID-19, nonhuman primate, AIDS, immunodeficiency

## Abstract

People living with HIV (PLWH) have an increased risk of severe COVID-19, including prolonged viral shedding and emergence of mutations. To investigate the simian immunodeficiency virus (SIV) macaque model for HIV/SARS-CoV-2 coinfection, seven SIV+ rhesus macaques were co-infected with SARS-CoV-2. COVID-19 in all macaques was mild. SARS-CoV-2 replication persisted in the upper, but not the lower respiratory tract for 14 days post-infection. Animals showed impaired generation of anti-SARS-CoV-2 antibodies and T-cells. Animals also displayed transient changes in microbial communities in the upper airway and gastrointestinal tract. Evidence of SARS-CoV-2 evolution was observed in the upper respiratory tract. This study demonstrates that SIV/SARS-CoV-2 coinfection in rhesus macaques recapitulates aspects of COVID-19 in PLWH. We show that SIV impairs anti-SARS-CoV-2 immunity, potentially leading to prolonged viral shedding, altered pathogenesis, and viral evolution. This highlights the importance of HIV status in COVID-19 and supports the use of this model for HIV/SARS-CoV-2 coinfection.

## 1 Introduction

HIV infection is a risk factor for complications of SARS-CoV-2 infection, including severe COVID-19, post-acute sequelae of SARS-CoV-2 infection (PASC), and increased mortality (1, 2). Additionally, immunosuppressed or untreated HIV infection is shown to contribute to SARS-CoV-2 viral persistence and intrahost evolution (3), which likely extends the viral transmission window and may serve as a potential source for the emergence of variants of concern (VOC). Furthermore, individuals with low CD4 counts or unsuppressed HIV have weaker immune responses to COVID-19 vaccination and to natural SARS-CoV-2 infection (4–10). As a result, nearly half of hospitalized breakthrough cases occur in immunocompromised individuals (11). The risk of COVID-19 related hospitalization and death is consistently higher in those with low CD4 counts and in those with unsuppressed HIV due to drug resistance, or in PLWH not taking antiretroviral therapy (ART). Furthermore, in resource-limited settings access to ART is often limited, with approximately 25% of PLWH not receiving ART. Many studies evaluating COVID-19 in PLWH exclude individuals who are immunosuppressed or not on ART, which limits the generalizability of these findings. Developing a model to understand SARS-CoV-2 pathogenesis during uncontrolled HIV infection is crucial to gain insight into host immune factors involved in SARS-CoV-2 viral pathogenesis during immunosuppression.

There are several challenges in studying SARS-CoV-2 pathogenesis and COVID-19 in humans that can be addressed using animal models. SARS-CoV-2 infection in humans causes a wide range of physiological outcomes and capturing early virological and immunological events is challenging especially in asymptomatic individuals, those with mild symptoms, or vulnerable individuals with limited access to medical care. Additionally, many clinical studies are restricted to measurements in the blood and upper respiratory tract (e.g., nasal swabs). Although these sites are useful for detecting viral replication, they limit our ability to fully understand persistence at primary (e.g., lung) and secondary (e.g., gut mucosa) tissue sites of pathogenesis, which may harbor distinct viral populations (12). Because the initial immune responses to COVID-19 primarily occur in tissue, the lack of paired tissue and blood sampling in humans limits our understanding of the complexities of SARS-CoV-2 infection, evolution, and the host immune response. Animal models overcome this limitation by allowing longitudinal sampling from multiple tissue sites throughout infection.

Here, we established a rhesus macaque model of SARS-CoV-2 co-infection during untreated simian immunodeficiency virus (SIV)-induced immunosuppression. Using this model, we provide evidence for persistent SARS-CoV-2 infection, impaired anti-viral immunity, alterations to the microbiome, and intrahost SARS-CoV-2 viral evolution in immunocompromised rhesus macaques.

## 2 Materials and methods

### 2.1 Rhesus macaques and study design

Seven female rhesus macaques (aged 7–10 years, 5.9-9.9kg) were used. All animals were experimentally infected with SIVmac251 and subsequentially experimentally infected with SARS-CoV-2 (WA-1). Animals were housed at BIOQUAL, Inc. (Rockville, MD), an American Association for the Accreditation of Laboratory Animal Care International (AAALAC) accredited facility. All animal procedures were approved by the BIOQUAL Institutional Animal Care and Use Committee (IACUC) (IACUC #22-037P). Blood, bronchoalveolar lavage (BAL), stool, and nasal, tracheal, and rectal swabs were collected prior to and every 3–4 days post-SARS-CoV-2 infection (DPI). Sample collection occurred under ketamine sedation. Physical exams were conducted at each sampling timepoint including body weight, body temperature, and clinical scoring (**Supplementary Tables 1**-**3**).

### 2.2 Sample collection and processing

Blood was collected by femoral venipuncture using a vacutainer 21g x 1” blood collection needle or Abbott Butterfly 23g x ¾” tubing attached to a vacutainer evacuated blood collection tube holder and tube. The volume of blood withdrawn did not exceed guidelines with respect to the animal’s body weight and physical condition. BAL was collected as previously described (13). 10mL of saline was flushed and retrieved through the tube. Swabs were collected using Copan flocked swabs, placed in PBS or viral transport medium (Lampire Biological Laboratories, Inc, Pipersville, PA), and stored at <-70°C.

Blood was collected in BD vacutainer EDTA tubes and centrifuged to isolate plasma. Peripheral blood mononuclear cells (PBMC) were further isolated from remaining blood using Histopaque^®^-1077 (Millipore Sigma, Burlington, MA). After centrifugation, PBMC were carefully removed. Red blood cells, if visible, were removed using ACK lysing buffer. PBMC were counted using a Nexcelom cellometer (Nexcelom Bioscience, Lawrence, MA). Cells were resuspended in freezing media (FBS + 10% DMSO) and stored at -75 to -80°C for 12–24 hours before transfer to liquid nitrogen. Serum was isolated by centrifugation of blood collected into BD Vacutainer^®^ SST™ tubes.

At necropsy, lung tissue was collected from all 5 lobes and stored for histopathology or snap-frozen on dry ice for viral quantification.

### 2.3 SIV and SARS-CoV-2 viral infections

Rhesus macaques were challenged 1–5 times intravaginally with a low dose of SIVmac251 (1:25 dilution, 800 TCID50) to model natural SIV infection. Four to eight months post-SIV acquisition, animals were co-infected intranasally (0.5mL/naris) and intratracheally (1.0mL) with a total of 1.3 x 10^6^ TCID_50_/mL SARS-CoV-2, isolate USA-WA1/2020 (NR-53872, BEI Resources, Manassas, VA). A full table of animal characteristics at the time of SARS-CoV-2 co-infection is given in **Supplementary Table 2**.

### 2.4 Clinical disease monitoring

Body weight, rectal temperature (**Supplementary Figure 1**), and awake and sedated clinical scoring was recorded in **Supplementary Tables 1**, **3**. Complete blood counts and serum chemistries were quantified via BD TruCount at -7, 3, 7, and 14 DPI (**Supplementary Tables 4, 5**). SIV viral RNA was isolated using the Qiagen QIAsymphony DSP Virus/Pathogen Midi Kit (96)/QIAgility (QIAGEN, Cat #937055), and levels were evaluated using the Applied Biosystems StepOne Plus Quantitative Real-Time PCR (Applied Biosystems, Waltham, MA). The limit of quantification (LOQ) for this assay is 62 RNA copies/mL (1.7log_10_) in 0.5mL of plasma.

### 2.5 Quantification of SARS-CoV-2 viral and subgenomic RNA

Viral RNA was isolated using the Qiagen MinElute Virus Kit (QIAGEN Cat #57704, Germantown, MD) per the manufacturer’s instruction and quantified by optical density at 260nm. SARS-CoV-2 viral RNA was assessed in the BAL, nasal swabs, tracheal swabs, and rectal swabs collected at 3, 5, 7, 10 and 14 DPI. cDNA synthesis was performed using the SensiFAST Probe Lo-ROX One-Step Kit (Meridian Life Science, Cat #78005, Memphis, TN) and the following primer/probe sequences: 2019-nCoV_N1-F:5’-GAC CCC AAA ATC AGC GAA AT-3’, 2019-nCoV_N1-R: 5’-TCT GGT TAC TGC CAG TTG AAT CTG-3’, 2019-nCoV_N1-P: 5’-FAM-ACC CCG CAT TAC GTT TGG TGG ACC-BHQ1-3’. All samples were tested in triplicate, and all standard curves were tested in duplicate. cDNA was then amplified via qPCR using the Applied Biosystems 7500 Real-Time PCR System (Applied Biosystems, Cat # 4351104, Foster City, CA) and was cycled at 48°C for 30 minutes then 95°C for 10 minutes, followed by 40 cycles at 95°C for 15 seconds and 55°C for one minute. RNA copies/mL were calculated by extrapolating the standard curve and multiplying by the reciprocal of 0.05mL extraction volume, giving a detection range of 50 – 5x10^8^ copies/mL. Subgenomic-N (Sg-N) and Subgenomic-E (Sg-E) RNA was quantified using previously described methods (14).

### 2.6 Infectious viral load assay (TCID_50_)

Vero TMPRSS2 cells (NIAID Vaccine Research Center, Bethesda, MD) were plated at 25,000 cells/well in DMEM + 10% FBS + gentamicin and incubated at 37°C with 5.0% CO_2_. Once 80-100% confluency was reached, medium was replaced with DMEM + 2% FBS + gentamicin. Cells were plated at 10-fold dilutions, along with virus of known titer and medium-only wells. The plates were incubated at 37°C with 5.0% CO_2_ for 4 days. Cell monolayers were visually inspected for cytopathic effect (CPE). TCID_50_ values were calculated using the Read-Muench method with a limit of detection of 2.7 log_10_ TCID50/mL.

### 2.7 Multiplex immunoassay

Cytokine and chemokine levels in plasma and BAL were analyzed using the Cytokine & Chemokine 30-Plex NHP ProcartaPlex™ Panel (ThermoFisher Scientific), per the manufacturer’s instruction. The quantification of analytes was assessed on a Bio-Plex 200 system (BioRad), and plasma concentrations were determined from a standard curve using 5-PL logistic regression. Heatmap visualizations were created using the pheatmap method from the ComplexHeatMap (v.3.2) package. Each day is independently clustered using the default pheatmap settings with the clustering distance method set to Euclidean and the clustering method set to complete.

### 2.8 H&E lung pathology and scoring

Histopathology was performed on H&E-stained lung sections from seven nonhuman primates. Five lung sections (right upper, middle, lower and left upper and lower) from each animal were evaluated and scored for histopathologic lesions on a scale from absent (0) to severe (4) for five histopathologic lesions. In addition to semiquantitative scores, quantitative measurement was performed using a deep learning algorithm trained to quantify all types of inflammation (HALO AI, Indica Labs) and reported as percentage of the lung section with inflammation. The results of the algorithm were correlated with the semiquantitative scores of the pathologist. Total inflammation for each animal was determined by summating the percentage of inflamed lung across all five sections available for all animals (minimal <5%, and mild inflammation 5-10%). A summation of the gross pathology and histological findings can be found in **Supplementary Tables 6**, **7**.

### 2.9 MPO lung histology

4µm tissues sections of lung were mounted on Superfrost Plus microscope slides, baked for 3 hours at 60°C and passed through xylene, graded ethanol, and double distilled water to remove paraffin and rehydrate tissue sections. A microwave was used for heat induced epitope retrieval (HIER). Slides were boiled for 16 minutes in a Tris-based solution, pH 9 (Vector Labs H-3301), containing 0.1% Tween20. Slides were briefly rinsed in hot, deionized water and transferred to a hot citrate-based solution, pH 6.0 (Vector Labs H-3300) where they were allowed to cool to room temperature. Slides were removed from the antigen retrieval solution, washed in phosphate-buffered saline, deionized water, and Roche reaction buffer before being loaded on the Ventana Discovery Ultra Autostainer where they would undergo blocking, primary antibody (rabbit anti-MPO, Dako A0398, 1:1000 dilution) incubation, washing, secondary antibody incubation, washing, DAB color development, and counterstaining with hematoxylin II. Upon removal, slides were put through alternating manual washes of deionized water containing 0.1% Dawn dish soap and plain deionized water for a total of 5 cycles. Slides were then cleared in ethanol (80%, 95%, 100%, 100%) and three xylene changes before being permanently mounted with StatLab Acrymount Mounting Media. After drying overnight, slides were digitally imaged at 40X with a Hamamatsu NanoZoomerS360. Information regarding control samples can be found in **Supplementary Table 8**.

### 2.10 IL-4/IFN-γ enzyme-linked immunospot assay

Antigen-specific PBMC secreting IFN-γ or IL-4 were measured using the Human IFN-γ/IL-4 Double-Color ELISPOT Kit (ImmunoSpot, Shaker Heights, Cleveland, OH), per the manufacturer’s protocol. PBMC were stimulated for 48 hours with SARS-CoV-2 peptide pools (17-, 13-, or 12-mers, with 10 amino acid overlaps) (BEI Resources, Cat # NR-52402, NR-52403, NR-52404, NR-52405, Manassas, VA) at a concentration of 1µg/mL per peptide. DMSO, and PMA and ionomycin (ThermoFisher) were used as negative and positive controls, respectively. Spots were counted on an Immunospot Analyzer with CTL Immunospot Professional Software (Cellular Technology Ltd. Sharker Heights, Cleveland, OH). Spot forming cells (SFCs) in peptide stimulated wells were computed following subtraction of SFCs detected in DMSO stimulated controls wells and were considered positive if the number of SFC was > 4 spots per 1x10^5^ plated cells.

### 2.11 SARS-CoV-2 binding antibody ELISA

Antigen-specific IgG and IgM responses were detected in sera by ELISA using recombinant SARS-CoV-2 Spike protein (Sino Biologicals, Cat #40589-V08B1, #40589-V08H28, #40589-V08H33) as the capture antigen. Information regarding control sera can be found in **Supplementary Table 9**. ELISA plates (Corning Inc, Corning, NY) were coated with antigen (1µg/mL) or with serial dilution of purified NHP IgG (NHPRR, Boston, MA) in 0.1M phosphate-buffered saline (PBS). Consecutively, serially diluted sera, goat anti-monkey IgG-HRP (Invitrogen, Waltham, MA), SureBlue TMB (SeraCare, Milford, MA), and HCl were added. Plates were washed with 0.05% PBS/Tween20 in between the addition of sera, goat anti-monkey IgG-HRP, and SureBlue TMB. Absorbance values were read at 450 nm (ELx808, BioTek Instruments Inc., Santa Clara, CA). Concentrations were analyzed using a five-parameter (5-PL) standard curve interpolation on Prism 8.3.0 (GraphPad, San Diego, CA).

### 2.12 D614G SARS-CoV-2 Spike pseudovirus neutralization

Lentivirus-based pseudoneutralization assays based on the B.1 D614G strain were performed on specimens at the University of Washington Virology Lab as previously described (15). 96-well plates were seeded with 17,500 293T cells constitutively expressing ACE2 (293T-ACE2), in a final volume of 50µL D10 media (DMEM, 10% FBS, 1% Pen/Strep), and allowed to adhere overnight. Serum was diluted 10-fold in D10 media and then serially diluted 3-fold over six additional dilutions in 96-well U-bottom plate. The viral stock was diluted in D10 media to ~8 x 10^6^ RLU/mL and 60µL of diluted viral stock was added to 60µL of each serum dilution. The virus and serum were incubated for one hour at 37°C, and then 100µL of the mix was added directly to the pre-seeded 293T-ACE2 cells. Approximately 50–55 hours post-infection, 100µL of media was removed from the cells, 30µL of Bright-Glo (Promega) was added, plates were incubated for 2 minutes and RLU of each well was measured using a VICTOR Nivo Plate Reader (Revvity) with an integration time of one second. Each plate included wells infected with non-enveloped pseudovirus (NoVEP) to determine the background RLU and wells infected with virus without serum (no-serum) to determine the expected RLU without inhibition. RLU values in test wells are normalized to percent inhibition using the NoVEP and no-serum well averages. Pseudovirus neutralization assay results are reported as the 80% neutralizing dilution titers (ND80), based on four-parameter logistic regression analysis.

### 2.13 SARS-CoV-2 culture assay

Bronchoalveolar lavage (BAL) fluid and nasal, throat, and rectal swab samples collected 7 days pre-infection, and on 3 and 14 DPI were cultured to recover live SARS-CoV-2 virus. BAL and swab samples were placed in Teknova Viral Transport Medium (VTM), filter sterilized and inoculated onto Vero E6 cells expressing human angiotensin-converting enzyme 2 and transmembrane Serine Protease 2 (VeroE56AT cells), as previously described (16). Virus-positive cultures were collected (**Supplementary Table 10**) for whole genome viral sequencing.

### 2.14 SARS-CoV-2 viral sequencing and analysis

Viral RNA was extracted using Quick-RNA Viral Kit (Zymo Research) and cDNA synthesized with SuperScript IV (Invitrogen). PCR tiling was performed with the xGen SARS-CoV-2 Amplicon Panels. Libraries were prepped with TruSeq Stranded Total RNA kit and barcoded using the Nextera XT Index kit (Illumina). Pooled samples were purified with AMPure XP beads and sequenced on the Illumina NextSeq platform. FASTQ files were demultiplexed, trimmed with TrimGalore (v0.6.10), and mapped to SARS-CoV-2 reference genome SARS-CoV-2/human/USA/USA-WA-CDC-02982586-001/2020 (accession: MN985325.1) using bwa (v07.17). Primers were clipped with iVar (v1.4.2). Variants were called with samtools (v1.13) and filtered by minimum quality score of 20, and a minimum allele frequency of 0.03 with at least 20x depth using iVar. SNPs found in only one sample or those present in the inoculating virus and indels were removed.

### 2.15 Microbiome data and analysis

DNA was extracted from stool and rectal, nasal and tracheal swabs using QIAamp PowerFecal Pro DNA Kit (Qiagen). DNA elution volumes are as follows: stool in 50μL, rectal and nasal swabs in 30μL, and tracheal swabs in 25μL. Sterile Water was used as a negative control for stool extractions and a sterile cotton swab soaked in sterile water was used as a negative control for swab extractions. Amplicon libraries of the V3-V4 region of the 16S rRNA gene were prepared in accordance with Illumina’s recommendations for 16S Metagenomic Sequencing Library Preparation (Part# 15044223 Rev. B) using primers 347F and 803R (17). Libraries were sequenced on the Illumina NextSeq2000^®^ using the NextSeq 1000/2000 P1 Reagents with 600 cycle chemistries (Illumina). After demultiplexing, paired end sequences were imported into QIIME2 v. 2023.9.1 (18). Primers were trimmed using the cutadapt plugin (19). Denoising, quality filtering, and enumeration of amplicon sequence variants (ASVs) were performed using DADA2 (**Supplementary Table 11**) (20). Taxonomic assignments were established using scikit-learn naïve Bayes classifier trained on the SILVA SSU Ref NR99 138.1 dataset (**Supplementary Table 12**) (21). A phylogenetic tree for downstream analysis was constructed using SATé-Enabled Phylogenetic Placement (SEPP) with the SILVA 128 release reference tree (22). To control for any potential extraction contaminates or sequencing artifacts, the negative controls mentioned above were imported into QIIME2 and analyzed in conjunction with samples. The total number of ASVs found in negative controls was significantly lower than any sampling location and the identity of features found in the controls were noted (**Supplementary Figure 2**). Alpha and beta diversity was calculated in QIIME2 using the core phylogenetics metrics plugin with a rarefaction depth of 20,600 to retain all samples and exclude negative controls. The ASVs, taxonomy, and diversity metrics were imported into R (v. 4.4.1) using qiime2R (v. 0.99.6) for further analysis and visualization. Relative abundance percentages were calculated as the median relative abundance for samples with those taxa. Differential abundance testing was performed on unrarefied counts from imported feature tables using ANCOMBC2 (23) to identify genus level features changing over time. Genera with a prevalence <10% were excluded from analysis. Timepoints were treated as a categorical variable to allow for multigroup analysis. Animal ID was considered as a random intercept, with sensitivity and structural zero analysis set as true. The p-adjustment method was set to the Benjamini-Hochberg procedure to control the false discovery rate (**Supplementary Table 13**). All microbiome related plots were made with ggplot2 (v. 3.5.1).

### 2.16 ELISAs for inflammation and gut integrity

Concentrations of myeloperoxidase (MPO) were detected in undiluted plasma by enzyme-linked immunosorbent assay (ELISA) using the Human Myeloperoxidase DuoSet ELISA (R&D Systems, Inc, Minneapolis, MN), per manufacturer’s protocol. Diluted plasma was used to determine the concentrations of soluble CD14 (sCD14) (1:200) using the CD14 Human ELISA Kit (ThermoFisher, Waltham, MA), C-reactive protein (CRP) (1:1000) using the Monkey CRP ELISA, CRP-3 (Life Diagnostics, West Chester, PA), and intestinal fatty acid binding protein (IFABP) (1:2) using the Monkey IFABP/FABP2 ELISA Kit (MyBiosource, San Diego, CA).

### 2.17 Statistical analysis

Non-parametric statistical methods were used for all comparisons, unless otherwise noted. Specifically, the Friedman test was used for complete data or the Kruskal-Wallis test was used for incomplete data, with Dunn’s multiple comparison tests used for comparisons across timepoints and Kruskal-Wallis tests used to compare values across groups. All analyses were conducted using two-sided tests with an alpha value of 0.05. Analyses were conducted in Prism version 10.3.1 (GraphPad). Statistical analysis for microbiome related figures was performed in R (v. 4.4.1) using the rstatix (v. 0.7.2) package for the Friedman test and Dunn’s multiple comparisons.

## 3 Results

### 3.1 SARS-CoV-2 viral replication persists in the upper respiratory tract, but not the lower respiratory tract of SIV+/SARS-CoV-2+ co-infected rhesus macaques

**Figure 1:**
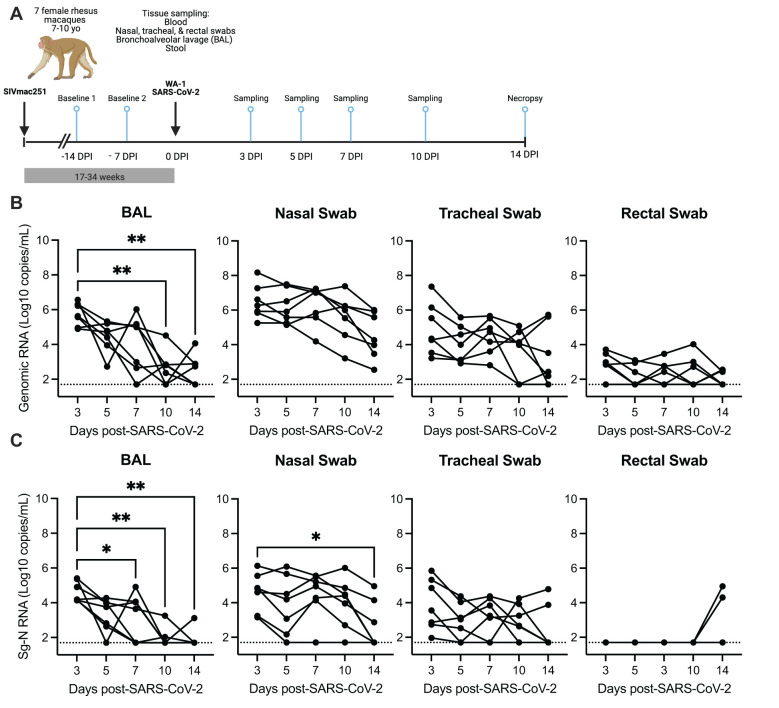
SARS-CoV-2 viral replication persists in the upper, but not the lower respiratory tract in SIV-infected macaques 14 days after infection. **(A)** Seven female rhesus macaques were experimentally challenged with SIVmac251 and then co-infected with SARS-CoV-2 (WA1/2020) and followed for 14 days. Tissue sampling and clinical exams occurred prior to SARS-CoV-2 infection and on days 3-, 5-, 7-, 10-, and 14-days post SARS-CoV-2 infection (DPI). Created in https://BioRender.com. Quantification of SARS-CoV-2 **(B)** viral RNA or **(C)** subgenomic-N viral RNA as determined by qRT-PCR. The dotted line indicates the lower limit of detection of the assay (50 copies/mL). **(B, C)** Friedman test with Dunn’s *post hoc* test versus 3 DPI, * p < 0.05, ** p < 0.01.

Seven female rhesus macaques, aged 7–10 years, were previously infected with SIVmac251 by repeat low dose intravaginal challenge (**Supplementary Table 2**). All animals were co-infected 17–32 weeks (4–8 months) post-initial SIV infection with 1.3 x 10^6^ TCID_50_/mL SARS-CoV-2 (USA-WA1/2020) and monitored for 14 days post infection (DPI) (**Figure 1A**; **Supplementary Table 2**). At the time of co-infection, the median SIV viremia was 5.00 (4.08-6.03) log_10_ copies/mL of plasma (**Supplementary Figure 3**). All animals had signs of immunosuppression including depleted peripheral CD4 counts (<500 cells/µL of blood) in 4/7 (57%) of animals (median 494 (274-1090) cells/µL of blood) and a blood CD4/CD8 ratio of <1, a biomarker of HIV/AIDS disease progression, in 7/7 (100%) of animals (**Supplementary Figure 3**, **Supplementary Table 2**) (24). Mild anemia, hyperglycemia, thrombocytopenia, and lymphopenia were also noted in a few of the animals (**Supplementary Table 5**).

Longitudinal SARS-CoV-2 viral burden was evaluated in bronchoalveolar lavage (BAL), respiratory mucosal secretions (nasal and tracheal swabs), and rectal swabs by qRT-PCR evaluation of genomic viral and subgenomic (Sg) RNA (N and/or E) and infectious virus was detected by TCID50 assay (**Figures 1B, C**; **Supplementary Figures 4**, **5**). At 3 DPI, robust viral replication was detected in all animals in the upper (nasal and tracheal swabs) and lower respiratory tract (BAL), with lower and variable viral levels detected in rectal swabs (**Figures 1B, C**; **Supplementary Figure 4**). Infectious virus was detected in 6/7 (86%) animals at 3 DPI in the BAL and nasal swabs, in 1/7 (14%) animals in the tracheal swab and in none of the rectal swabs (**Supplementary Figure 5**). Genomic RNA in rectal swabs was detected in 5/7 (72%) animals at 3 DPI, but only in 3/7 (43%) by 14 DPI (**Figure 1B**). Interestingly Sg-N RNA was not initially detected in rectal swabs in any of the animals for the first 10 days of the infection but was detected in 2/7 (29%) of animals at 14 DPI (**Figures 1B, C**). Genomic and subgenomic levels of RNA (Sg-N and Sg-E) and infectious virus significantly decreased in the BAL 10–14 DPI, with the detection of viral RNA in 3/7 (43%) and Sg-N RNA in 1/7 (14%) of animals by 14 DPI and is evidence for viral clearance in the lung (**Figures 1B, C**; **Supplementary Figure 4**). In contrast, only the levels of Sg-N RNA significantly decreased in nasal swabs at 14 DPI (**Figure 1C**), but the persistence of genomic (7/7, 100%) and Sg-E viral (3/7, 43%) RNA was evident at 14 DPI (**Figure 1B**; **Supplementary Figure 4**). These results were further confirmed by the detection of infectious virus in the nasal swabs in 3/7 (43%) animals at 14 DPI but in none of the BAL specimens (**Supplementary Figure 5**).

Virus persistence was also evident in the tracheal swabs; genomic RNA was detected in 5/7 (71%) and Subgenomic-N RNA in 2/7 (29%) animals at 14 DPI (**Figures 1B, C**). Collectively, these data suggest that SARS-CoV-2 may persist longer in the upper respiratory tract of SIV+ animals. To investigate this hypothesis, we next compared the levels of SARS-CoV-2 RNA at 7–10 DPI from our study to data sets from two published studies of naïve rhesus macaques of similar age who received a comparable WA-1 SARS-CoV-2 infection, but were necropsied earlier at 10 DPI (25, 26). The levels of genomic RNA in the nasal swabs, but not the BAL, tracheal or rectal swabs, were significantly higher at 7 and 10 DPI in our SIV+/SARS-CoV-2+ co-infected animals in comparison to SIV- animals infected with SARS-CoV-2 (**Supplementary Figure 6**) (26). Similarly, we compared the levels of subgenomic-E RNA in the BAL and nasal swabs collected in our study with results from those from Chandrashekar et al. (25), and found that the levels of Sg-E were significantly higher in the nasal swabs, but not the BAL, of SIV+ versus naïve animals at 10 DPI (**Supplementary Figure 6**). Collectively, these data provide evidence that SIV infection may contribute to persistence or delayed clearance of SARS-CoV-2 virus in the upper, but not the lower, respiratory tract.

### 3.2 Mild COVID-19 disease progression during SIV+/SARS-CoV-2+ co-infection

We next evaluated whether SIV-induced immunosuppression had an impact on SARS-CoV-2 disease pathogenesis. SARS-CoV-2 clinical disease was generally mild in all animals as measured by transient increases in body temperature, and blinded clinical scoring throughout infection, however significant declines in body weight were detected at 7 and 14 DPI (**Supplementary Figure 1**, **Supplementary Table 1**, **3**). Elevated serum levels of alanine aminotransferase (ALT) and decreased serum levels of hemoglobin were also noted 1–2 weeks after infection (**Supplementary Figure 1**, **Supplementary Table 5**). Levels of SIV plasma viremia were unchanged by SARS-CoV-2 infection and there were transient dips in the levels of peripheral CD8 cells at 3 DPI that returned to pre-SARS-CoV-2 levels by 7 DPI (**Supplementary Figure 3**), indicating SARS-CoV-2 infection did not further promote peripheral SIV disease progression.

**Figure 2:**
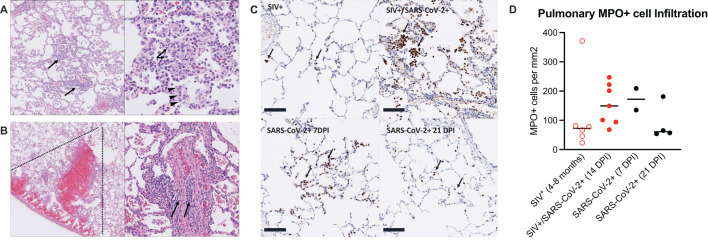
Mild pulmonary inflammation and pulmonary infarction in SIV+/SARS-CoV-2+ rhesus macaques. **(A)** Histopathology of SARS-CoV-2 infection. Animal T985, right middle lung. Left Panel: The pulmonary interstitium is multifocally infiltrated by low to moderate numbers of mixed inflammatory cells (arrows). Right Panel: The inflammatory infiltrate is composed predominately of histiocytes with fewer numbers of lymphocytes and neutrophils (arrows). Inflamed alveolar septa are segmentally lined by type II pneumocytes (arrowheads). **(B)** Pulmonary infarction. Animal T982, right lower lung. Left Panel: There is wedge-shaped (dotted lines) pulmonary hemorrhage consistent with infarction effecting a focal region of the right lower lobe. Right Panel: Vessels within the region of infarction exhibit medial and intimal expansion by low to moderate numbers of lymphocytes (arrows). **(C)** Immunohistochemistry for myeloperoxidase (MPO). Top left: Animal EE87, chronic SIV infection. Low numbers of MPO positive cells (brown, arrows) are present within alveolar septa and alveoli. Top right: Animal T985, SIV+/SARS-CoV-2+ co-infection, 14 DPI. There is patchy infiltration of alveoli and alveolar septa with aggregates of MPO+ cells. Bottom left: Animal LM74, SIV-/SARS-CoV-2+, 7 DPI. Alveolar septa are multifocally expanded by small aggregates of MPO+ cells. Bottom right: Animal KE93, SIV-/SARS-CoV-2 + 21 DPI. Low numbers of MPO positive cells are present within alveolar septa and alveoli. Bar=100 μm. MPO-DAB. **(D)** MPO immunohistochemistry quantification. Historical control specimens: SIV+ (n=6), SARS-CoV-2+ (n= 2, 7 DPI; n=4, 21 DPI). Medians are shown. Kruskal-Wallis test between groups showed no significant differences.

At necropsy, lung inflammation and pathology were evaluated (**Figure 2A**; **Supplementary Table 7**). All animals exhibited minimal to mild pulmonary inflammation. Two animals, T985 and T986, exhibited minimal to mild pulmonary inflammation, and the remaining five animals exhibited minimal pulmonary inflammation with modest differences between them. Type II pneumocyte hyperplasia, a common finding in SARS-CoV-2 infection, was closely associated with inflammation, indicating that SARS-CoV-2 infection was likely the driver of pulmonary inflammation observed in these animals. Type II pneumocyte hyperplasia was observed less frequently than pulmonary inflammation which may indicate resolving disease in these animals. Two animals had thrombotic lesions (T981 & T982): T982 had multiple pulmonary infarcts (**Figures 2B, C**), whereas T981 had an intravascular thrombus but no infarction. Thrombocytopenia was evident in both animals prior to SARS-CoV-2 infection (**Supplementary Table 5**), indicating the SIV infection is likely the primary driver of thrombotic disease, however SARS-CoV-2 has been shown to result in thrombotic disease in both humans and NHP and therefore may synergistically contribute to thrombus formation in the context of SIV co-infection.

### 3.3 Mild and persistent peripheral and pulmonary inflammation during SIV/SARS-CoV-2 co-infection

COVID-19 disease is typically more severe in individuals with HIV and those with higher levels of inflammatory markers have more severe COVID-19 outcomes (27). Based on this, we next evaluated peripheral inflammation during SIV/SARS-CoV-2 co-infection. Transient increases in plasma concentrations of C-reactive protein (CRP), a marker of inflammation, occurred in most animals 3–7 DPI (**Supplementary Figure 7**). Similarly, concentrations of soluble CD14 (sCD14), a marker of monocyte/neutrophil activation, transiently increased in three animals 3–14 DPI (**Supplementary Figure 7**). Circulating concentrations of intestinal fatty acid binding protein (IFABP), a marker of impaired gastrointestinal integrity, also transiently increased in 3 animals 3–5 DPI (**Supplementary Figure 7**).

Systemic and pulmonary inflammation was further evaluated in the plasma and BAL by multiplex immunoassay relative to pre-SARS-CoV-2 infection levels. In the plasma, four animals had persistent systemic inflammatory profiles starting 3–7 DPI and persisting to 14 DPI (T982, T986, 12M273, and T981), one animal (T988) had an acute inflammatory profile 3–5 DPI, and the remaining two animals had no substantial increase in the plasma analytes tested (**Supplementary Figure 8**). The systemic inflammatory profile consisted of several cytokines and chemokines important for the recruitment and differentiation of innate (CCL2/MCP-1, CCL4/MIP-1β, CCL11/Eotaxin, and CXCL8/IL-8) and adaptive immune cells (CXCL9/MIG, CXCL10/IP-10, CXCL11/I-TAC, CXCL12/SDF-1α, and CXCL13/BLC). In the BAL, all animals had proinflammatory cytokine profiles starting at 3 DPI, with the strongest profiles observed in 4 animals (T981, T985, T982, and T986) (**Supplementary Figure 8**). These 4 animals additionally had acute and high concentrations of interferon alpha (IFNα) and IL-7 at 3 DPI. This proinflammatory profile was primarily driven by several key molecules: CCL11/Eotaxin, an eosinophil chemoattractant; CXCL11/I-TAC, which recruits activated T-cells; CXCL13/BLC, which recruits B-cells; CXCL/IL-88, a neutrophil chemoattractant; IL-6, an activator of humoral immunity; and IL-1RA, which can suppress inflammation and antiviral responses. Two of the animals (T986 and T985) with the strongest pulmonary inflammatory responses at 3 DPI had little peripheral inflammation, while the other 2 animals (T981, T982) also had the strongest peripheral inflammatory profiles. Although many of the cytokine and chemokine levels returned to baseline levels at 14 DPI, a few were sustained through 14 DPI and in some animals, cytokine levels started to increase. For example, the peak levels of CXCL8/IL-8 (T988, T985), CXCL11/I-TAC (T985), CXCL9/MIG (T982, 12M273, and T985), and CCL2/MCP-1 (T982, 12M273) were observed at 14 DPI in specific animals. These data demonstrate that in some SIV-infected animals, SARS-CoV-2 co-infection prompts an acute inflammatory response in the lungs, while in others there is persistent inflammation in the lung and periphery. These data suggest that diverse inflammatory profiles may contribute to altered SARS-CoV-2 pathogenesis.

Pulmonary recruitment and activation of neutrophils in humans and NHP occurs during mild and severe COVID-19 (26, 28), indicating that neutrophils are an important player in the anti-viral defense against SARS-CoV-2 infection, but neutrophils can also contribute to immunopathology. We first measured circulating levels of myeloperoxidase (MPO), a neutrophil granule and marker of inflammation, and found that several animals had elevated levels prior to SARS-CoV-2 infection and throughout co-infection, with transient increases observed in a few animals (**Supplementary Figure 7**). We next measured neutrophil activity in the lung tissue at necropsy, in comparison to SIV+ and SARS-CoV-2+ mono-infected historical control specimens (**Supplementary Table 8**) (29–32). MPO+ cells in the lung during SIV/SARS-CoV-2 co-infection were evident in all animals (**Figures 2C, D**). The number of MPO+ cells in the lung was similar or higher in SIV+/SARS-CoV-2+ co-infected animals when compared to control specimens from SIV+ animals that had similarly been infected with SIVmac251 for 4–8 months (**Figures 2C, D**). In comparison to lung specimens from historically SARS-CoV-2+ infected animals, the number of MPO+ cells in lungs from co-infected animals was more consistent with lungs from 7 DPI, a timeframe of greater inflammation, than at 21 DPI, a post-acute phase timepoint (**Figure 2D**). Collectively, this data suggests that SIV/SARS-CoV-2 co-infection may promote the continual recruitment of inflammatory neutrophils to the lung following viral clearance from the lung.

### 3.4 SIV infection impairs the generation of anti-SARS-CoV-2 humoral and cellular immunity

**Figure 3:**
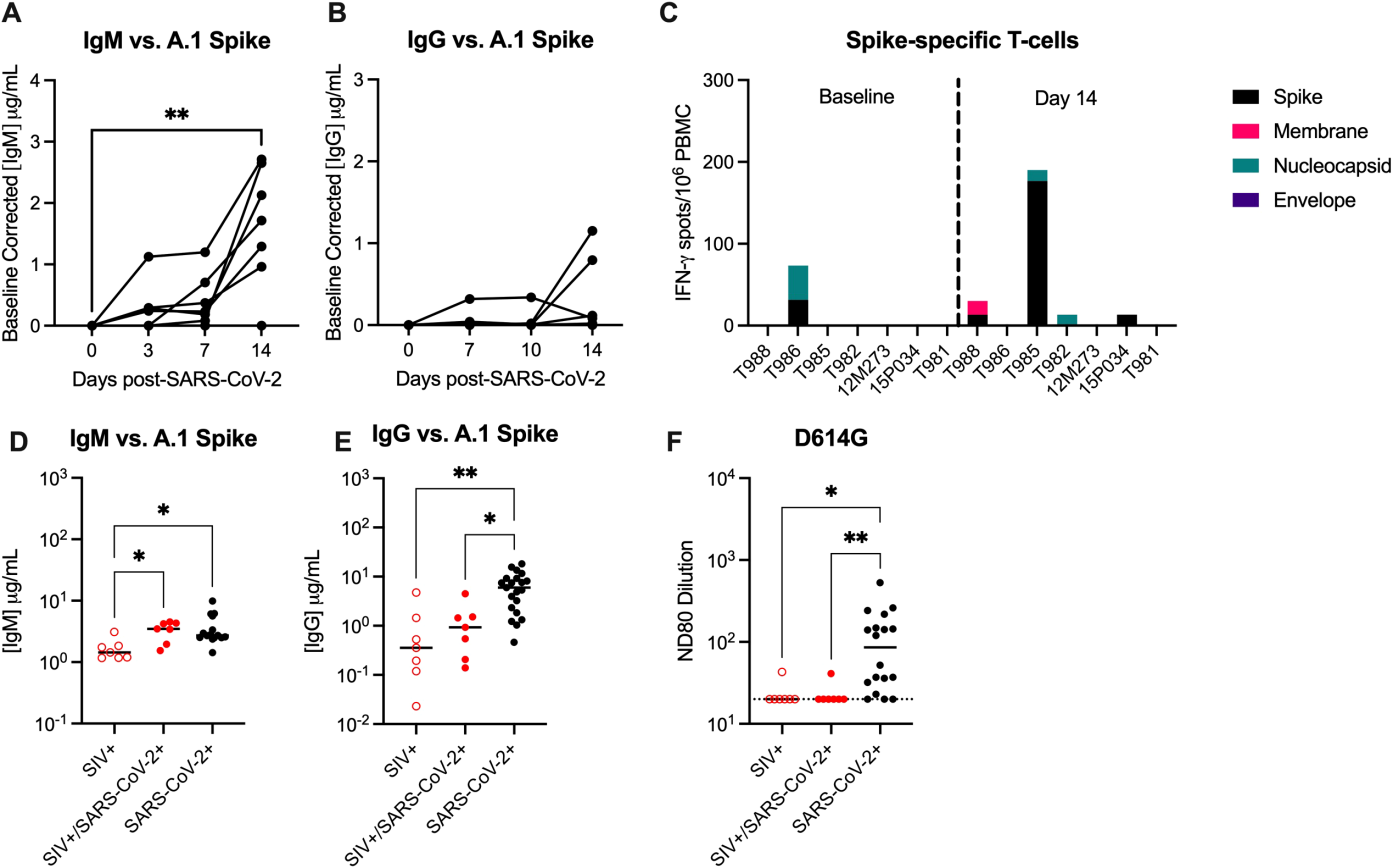
SIV infection impairs the generation of anti-SARS-CoV-2 immunity. Serum **(A)** anti-IgM and **(B)** anti-IgG enzyme linked immunosorbent assays (ELISAs) against A.1 Spike proteins. **(C)** Magnitude of IFN-γ T-cell responses were measured by ELISpot assay in PBMCs following 48-hour stimulation with overlapping peptide pools encompassing the WA.1 SARS-CoV-2 spike (S), membrane (M), nucleocapsid (N) and envelope **(E)** proteins. Comparative serum **(D)** anti-IgM and **(E)** anti-IgG ELISAs against A.1 Spike proteins with control specimens (n=15-21). **(F)** Pseudovirus neutralization titers (ND80) against D614G with historical controls (n=18). Dotted line indicates the limit of detection for the assay (20). **(A, B)** Friedman Test with Dunn’s *post hoc* test versus baseline, * p < 0.05, ** p < 0.01. **(D-F)** Medians with interquartile ranges are shown. Kruskal-Wallis test between groups, * p < 0.05, ** p < 0.01.

Humoral and cellular immunity are important for control of SARS-CoV-2 infection and protection against re-infection. We next evaluated anti-SARS-CoV-2 Spike binding antibodies in the sera by ELISA. At 14 DPI, 6/7 animals developed peripheral IgM antibodies, but only 2/7 (28.6%) animals developed IgG antibodies, albeit low, against the A.1 Spike (**Figures 3A, B**). Cross-binding IgM and IgG antibodies to the Spike protein of more contemporary variants of concern (BA.2, BA.5) were only detected in a few animals (**Supplementary Figure 9**). There is evidence in humans for conserved and cross-reactive SARS-CoV-2 T-cell epitopes across variants, including Omicron, and suggest an important role of T-cells in the control of SARS-CoV-2 (33). We next evaluated IFN-γ and IL-4-producing T-cells by ELISPOT in response to stimulation with peptides against SARS-CoV-2 Spike (S), Membrane (M), Nucleocapsid (N) and Envelope (E) proteins. Moderate IFN-γ producing T-cell responses, predominantly against Spike, were only detected in a single animal at 14 DPI (**Figure 3C**). Very low numbers of IFN-γ producing T-cells, against S, M, and/or N proteins were detected in 3 animals, and IL-4 producing T-cells were not detected in any animal against any antigen (**Figure 3C**). These results are in contrast to published NHP studies in which robust peripheral antigen-specific T-cells are typically detected as early as 7 days post-SARS-CoV-2 in naïve rhesus macaques (34).

Studies in NHP demonstrate that infection of naïve rhesus macaques with SARS-CoV-2 infection results in seroconversion (i.e. detection of IgG antibodies) in most animals 10–14 DPI (35, 36). Therefore, we obtained day 14 serum specimens from control animals infected with WA.1 SARS-CoV-2 to be used as SIV-/naïve controls in our immune assays, the animal details can be found in **Supplementary Table 9** (25, 37). Binding IgG, but not IgM antibodies, against WA.1, BA.2. and BA.5 Spike were significantly lower in SIV+/SARS-CoV-2+ animals when compared to SIV-/SARS-CoV-2+ control specimens (**Figures 3D, E**; **Supplementary Figure 9**). Furthermore, only one SIV+/SARS-CoV-2+ animal developed a low neutralizing antibody (nAb) response against SARS-CoV-2, as measured by pseudovirus neutralization assay, and SARS-CoV-2+ control specimens produced significantly higher nAb responses when compared to SIV+/SARS-CoV-2+ animals (**Figure 3F**). Collectively, these results provide evidence that SIV-induced immunosuppression impairs and/or delays the generation of humoral and cellular anti-SARS-CoV-2 immunity, which may be important for viral clearance and necessary for protection against SARS-CoV-2 re-infection.

### 3.5 The composition of the tracheal microbiome significantly changes during acute SIV/SARS-CoV-2 co-infection

**Figure 4:**
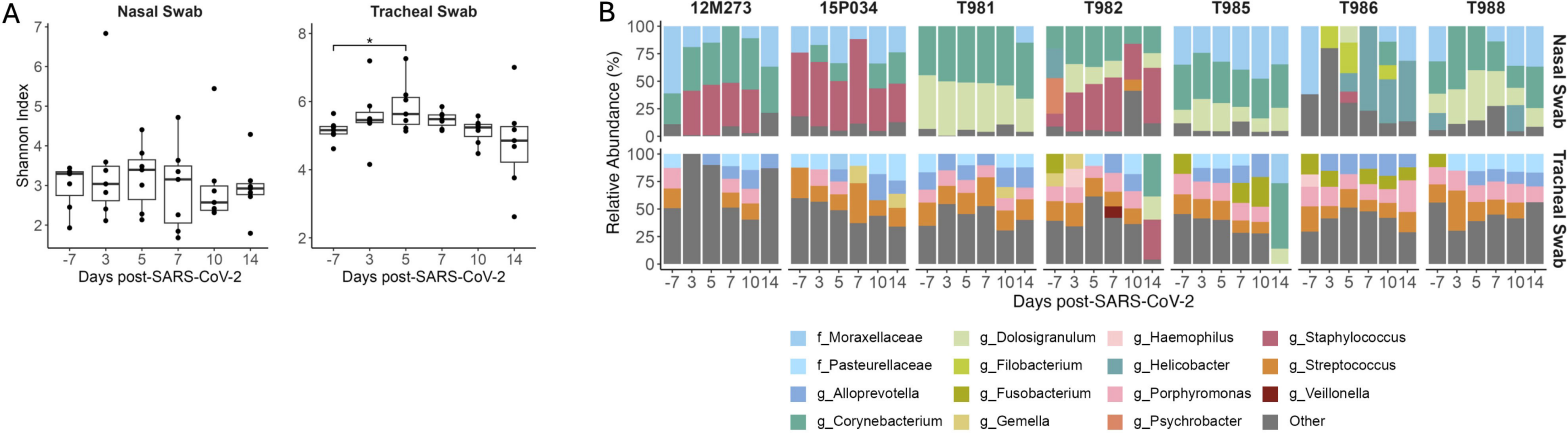
Transient changes in the tracheal microbiome during SIV+/SARS-CoV-2+ co-infection. **(A)** Shannon diversity of microbial DNA extracted from nasal and tracheal swabs. Medians with interquartile ranges are shown. The whiskers extend to the largest or smallest value no further than 1.5*IQR from the hinge. Friedman test of the Shannon diversity between timepoints. *Post hoc* Dunn Test for pairwise comparisons, * p < 0.05. **(B)** Relative Abundance of taxa classified to the family or genus level. Taxa that have an abundance of less than 10% in each sample are pooled into the “Other” category. Taxa that were only able to be classified to the family level have the prefix f_ while those classified to the genus level have the prefix g_.

SARS-CoV-2 and other respiratory diseases alter the oral, nasal, tracheal, and lung microbiomes, leading to more severe disease outcomes (38–42). Pulmonary diseases associated with HIV infection can also affect the respiratory microbiome (43). Therefore, we next evaluated compositional changes in the nasal and tracheal microbiomes during SIV+/SARS-CoV-2+ co-infection. The Shannon diversity index, a metric that combines measures of richness and evenness, was used to assess community-level changes over time. During SARS-CoV-2 infection in humans, Shannon diversity in the upper respiratory tract was shown to be greater in comparison to healthy individuals (38, 40). Shannon diversity significantly increased (p = 0.037) in tracheal swabs at 5 DPI when compared to pre-SARS-CoV-2 infection levels (**Figure 4A**) but was unchanged in nasal swabs at any timepoint post-SARS-CoV-2 co-infection. Overall community composition, which was measured using the unweighted UniFrac distance, showed the nasal and the tracheal swabs to have distinct microbial communities (**Supplementary Figure 2**). Therefore, we next determined the taxonomical composition of the airway (nasal and tracheal) microbiome within individual animals. Across all timepoints, the most abundant phyla in the nasal swabs were Firmicutes (39.90%), Actinobacteria (34.37%), Proteobacteria (17.51%), and Campylobacteria (15.5%); while the tracheal swabs were dominated by Firmicutes (37.13%), Bacteroidetes (29.74%), Proteobacteria (20.01%), and Fusobacteriota (10.67%) (**Supplementary Figure 10**), which is consistent with dominant phyla reported in NHP with pulmonary infections (44). At lower taxonomical levels, 12M273, 15P034 and T982 had a high abundance of *Staphylococcus* particularly after SARS-CoV-2 co-infection, while T981, T982, T985 and T988 had a notable presence of *Dolosigranulum* across timepoints (**Figure 4B**). The genus *Corynebacterium* (37.63%) and family Moraxellaceae (32.06%) appear in the nasal microbiome of most animals for at least one time point (7/7 and 6/7 respectively) (**Figure 4B**). The tracheal swabs were distinctly composed of the genera *Streptoccocus* (17.75%), *Porphyromonas* (15.03%), and *Alloprevotella* (14.61%) and the family Pasteurellacaea (14.22%) with all animals having similar genus level compositions. Four genera in nasal samples and three genera in tracheal samples showed significant differential abundance at a single timepoint after SIV+/SARS-CoV-2+ co-infection, but none differed consistently across multiple time points (**Supplementary Figure 11**). Overall, these findings suggest that acute SIV/SARS-CoV-2 co-infection causes transient changes in microbial diversity in the tracheal, but not the more uniform nasal microbiome.

### 3.6 SIV+/SARS-CoV-2+ co-infection induces genus level changes in the gut microbiome

**Figure 5:**
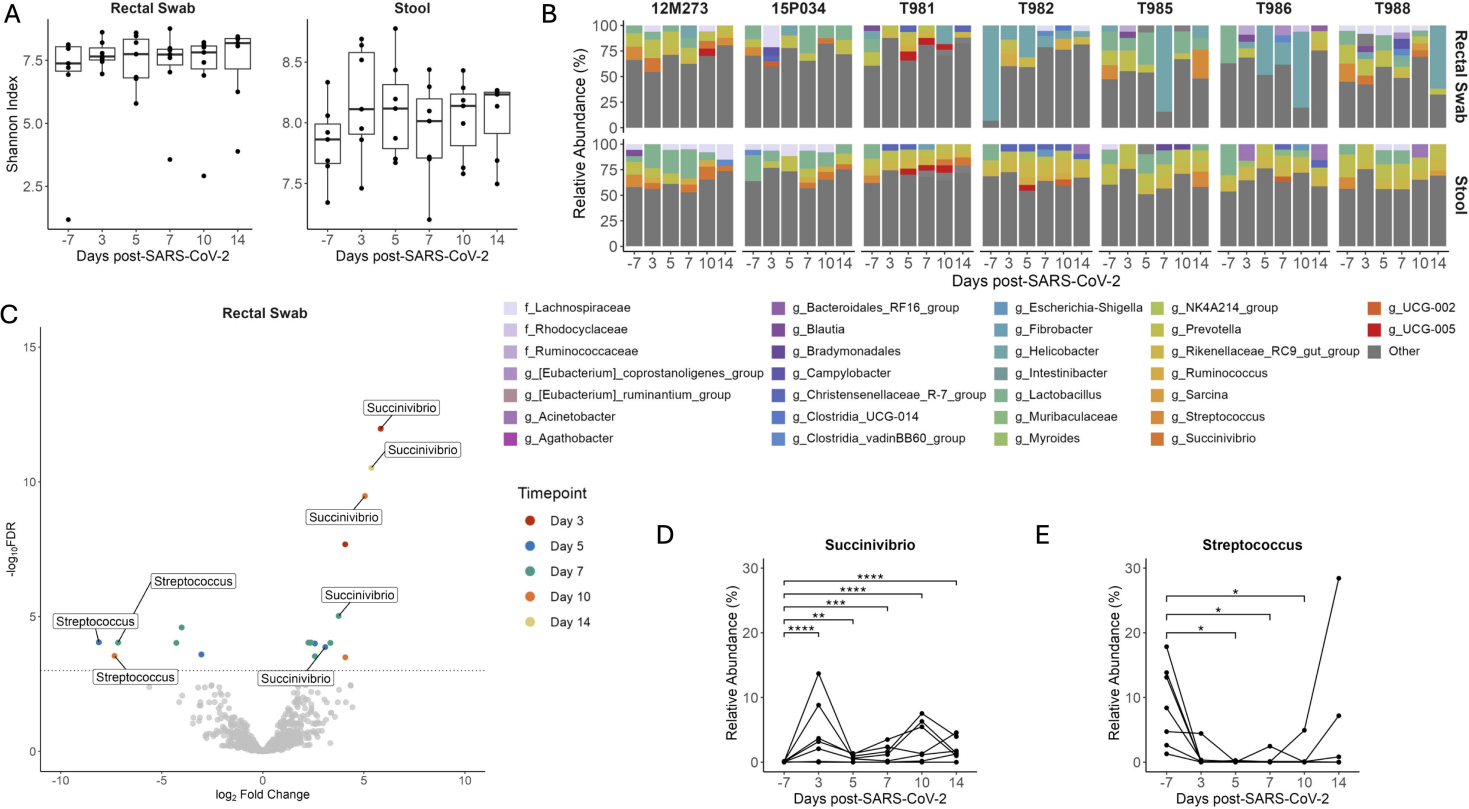
Changes in *Streptococcus* and *Succinivibrio* abundance in the gastrointestinal tract during SIV+/SARS-CoV-2. **(A)** Shannon diversity of microbial DNA extracted from rectal swabs and stool. Medians with interquartile ranges are shown. The whiskers extend to the largest or smallest value no further than 1.5*IQR from the hinge. Friedman test of the Shannon diversity between timepoints. **(B)** Relative abundance of taxa classified to the family or genus level. Taxa that have an abundance of less than 5% in each sample are pooled into the “Other” category. Taxa that were only able to be classified to the family level have the prefix f_ while those classified to the genus level have the prefix g_. **(C)** Differentially abundant genera relative to baseline in rectal swabs as determined by ANCOMBC2. Dotted line indicates a p adjusted value of < 0.05. Grey dots represent non-significant taxa, and colored dots represent taxa determined to be significantly abundant. Relative Abundance of **(D)** *Streptococcus* and **(E)** *Succinivibrio* in rectal swabs with p-adjusted values determined by ANCOMBC2, * p <0.5, ** p < 0.01, *** p < 0.001, **** p < 0.0001.

The importance of gut symbiosis and barrier function in reducing HIV disease progression is well described (45). Similar to HIV, SARS-CoV-2 replication occurs in the gastrointestinal (GI) tract (46) and promotes intestinal barrier dysfunction leading to bacterial translocation (47) and alterations to the gut microbiota (48, 49). To determine if SIV+/SARS-CoV-2+ co-infection altered the microbial community composition of the GI tract we first looked at changes in microbial diversity over time. Shannon diversity did not significantly change in stool or rectal swabs at any timepoint post-SARS-CoV-2 co-infection when compared to baseline (**Figure 5A**). The rectal swabs and stool had a high degree of similarity as shown by the unweighted unifrac distance, suggesting that these samples have analogous microbial niches (**Supplementary Figure 2**). We next looked at the longitudinal relative abundances of relevant taxa during SIV+/SARS-CoV-2+ co-infection. Taxonomic profiling of the gastrointestinal microbiome (stool and rectal swabs) showed Firmicutes (59.62%), Bacteroidetes (24.37%), Proteobacteria (4.54%) and, Campilobacterota (2.76%) to be the most abundant phyla across all animals at all timepoints (**Supplementary Figure 10**), which is consistent with previous findings (38). As expected for GI samples, most (80/84) had a high Shannon diversity index and were not dominated by a singular genus. On average, 98.25% of the taxa in each sample were comprised of taxa with a relative abundance of ≤5%. However, a subset of the rectal swabs (4/84) had low Shannon diversity and were dominated by *Helicobacter* (>50% abundance) (**Figures 5A, B**). The most abundant genera in both the stool and rectal swabs were *Lactobacillus* (13.57%), *Helicobacter* (12.29%), *Prevotella* (9.42%) and *Rikenellaceae_RC9_gut_group* (8.59%) (**Figure 5B**). Taxonomic changes between pre- and post- SARS-CoV-2 co-infection were determined using differential abundance analysis. In the rectal swabs and stool, 11 and 10 taxa, respectively were determined to be differentially abundant post-SARS-CoV-2 co-infection at a singular timepoint (**Supplementary Table 14**). *Succinivibrio* and *Streptococcus* were differentially abundant across multiple timepoints post-SARS-CoV-2 co-infection in both the rectal swabs (**Figure 5C**) and the stool (**Supplementary Figure 11**). *Succinivibrio* abundance was significantly higher in rectal swabs at all timepoints post-SARS-CoV-2 co-infection in comparison to baseline levels (**Figure 5D**). In contrast, *Streptococcus* abundance significantly decreased 5 DPI in rectal swabs relative to baseline and the abundance remained low in most animals for the 2-week period after co-infection (**Figure 5E**). *Streptococcus* abundance in rectal swabs of only two animals (T985 and 12M273) returned to levels at or above baseline at 14 DPI. Collectively, these data demonstrate that during the first two weeks of SIV+/SARS-CoV-2+ co-infection, there are significant changes in the abundance of specific genera, such as *Streptococcus* and *Succinivibrio*, within the gastrointestinal tract. However, these changes do not disrupt the overall community structure of the gut microbiome.

### 3.7 SIV infection may allow for intrahost SARS-CoV-2 viral evolution

**Figure 6:**
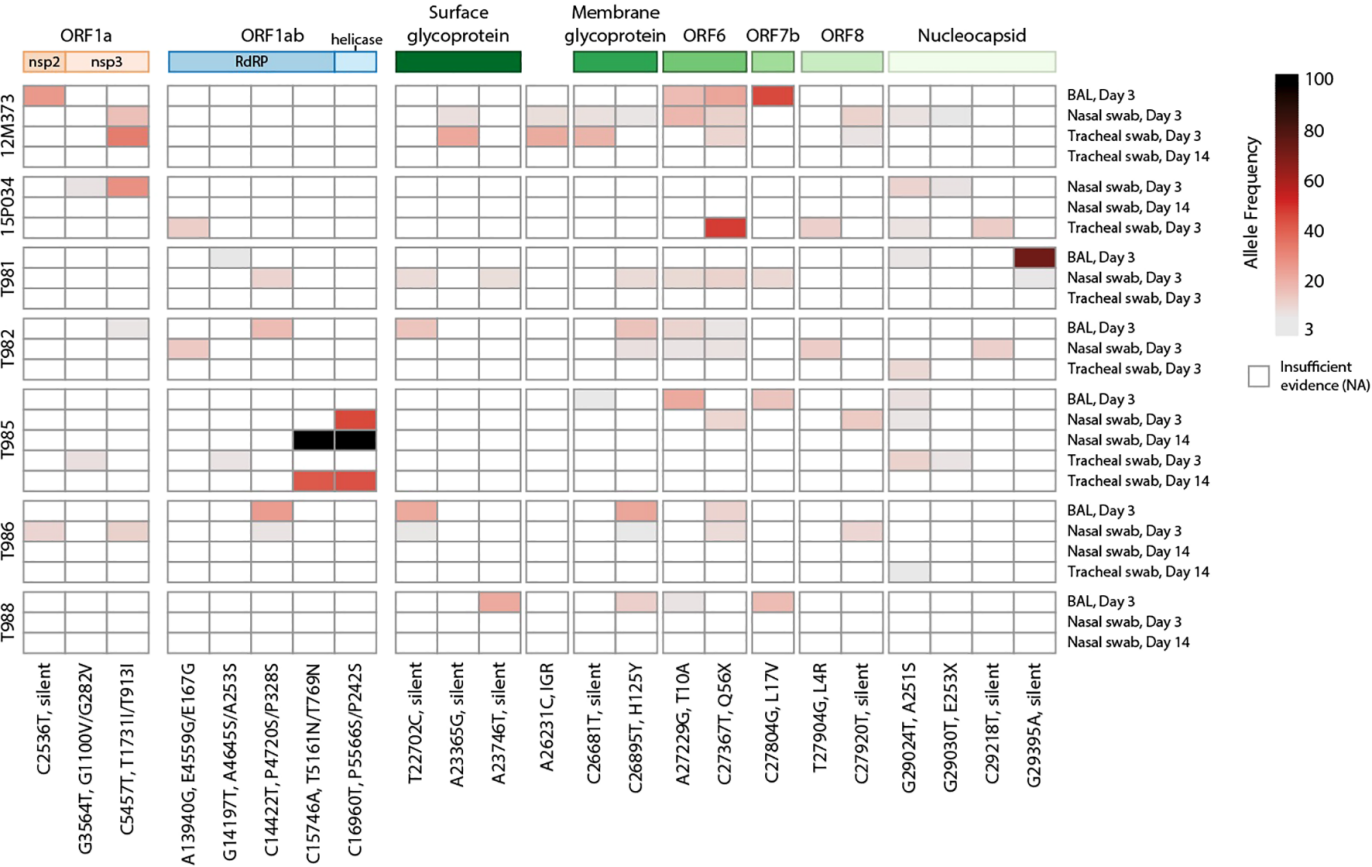
Heatmap of SARS-CoV-2 single nucleotide polymorphisms in SIV-infected macaques. Viral SNPs were identified against SARS-CoV-2/WA-1 reference (MN985325.1) Samples are shown in rows clustered by animal, with names on the left and sample type and timepoint on the right. SNPs are ordered in columns left to right, from 5’ to 3’ of viral genome. Allele frequency is represented by gradated red color in variants with a minimum of 3%. Variants with the highest (>80%) allele frequency are colored black. The genomic polyproteins and proteins associated with SNPs are annotated at the top of the heatmap. Mutations are labeled with the genomic nucleotide mutation, and, when applicable, the polyprotein and/or protein amino acid mutation. RdRP is RNA-dependent RNA polymerase, and IGR is an intergenic region.

Impaired cellular and humoral adaptive immunity during HIV contributes to poor SARS-CoV-2 viral clearance, providing more opportunity for SARS-CoV-2 viral evolution (50). This is a potential mechanism for the emergence of variants of concern (VOC) contributing to breakthrough immunity in vaccinated and pre-exposed individuals. SARS-CoV-2 persistence and intrahost evolution are reported in individuals with untreated or advanced HIV (3). We therefore wanted to determine whether SIV-induced immunosuppression similarly allows for intrahost SARS-CoV-2 evolution in an acute 2-week time period. BAL fluid and swabs (nasal, throat, and rectal) collected prior to SARS-CoV-2 challenge (-7 DPI) and on 3 and 14 DPI were cultured to recover live virus (**Supplementary Table 10**). Virus was not recovered from any of the pre-SARS-CoV-2 nor in any rectal swab cultures (3 or 14 DPI). Virus was also not recovered from BAL fluid on 14 DPI. Viral RNA was isolated from BAL and swab (nasal and throat) containing live SARS-CoV-2 virus and processed for whole genome sequencing. Viral sequences were mapped to SARS-CoV-2/WA-1/2020 clinical isolate to identify single nucleotide polymorphisms (SNPs). SNPs observed in ≥2 samples were identified in the ORF1a, ORF1ab, surface glycoprotein (S), S-ORF3a intergenic region, membrane glycoprotein (M), ORF6, ORF7b, ORF8 and nucleocapsid (N) genomic regions (**Figure 6**). A consistent pattern of mutations was observed across multiple animals and specimens (locations: M, H125Y; ORF6 T10A, Q56X; N, A251S). Two silent SNPs present in the inoculating virus at low frequencies (ORF1a/nsp3 C4897T at 5.2% and ORF8/T11C A27924C at 7.8%) were also enriched in samples in 6/7 and 7/7 animals, respectively (5.6-99.9%). Collectively, these data provide evidence that SIV-induced immune suppression may allow for intrahost SARS-CoV-2 viral evolution.

## 4 Discussion

This pilot study demonstrates the utility of the rhesus macaque HIV/AIDS model in understanding the underlying mechanisms contributing to COVID-19 in PLWH and in other immunocompromised individuals. Key findings confirmed or newly identified in this study using this model include evidence for 1) persistent SARS-CoV-2 virus replication, 2) systemic and pulmonary inflammation, 3) impaired anti-SARS-CoV-2 immunity, 4) alterations to the airway and gastrointestinal microbiome, and 5) intrahost SARS-CoV-2 viral evolution. Surprisingly, despite these factors, SARS-CoV-2 lung disease was mild and showed little immunopathology, a finding that is similar in SIV/SARS-CoV-2 co-infected pigtail macaques and SARS-CoV-2 infected rhesus macaques depleted of T-cells (51).

SARS-CoV-2 viral shedding is more persistent in PLWH (CD4 counts <200 cells/µL and/or high HIV viral load) in comparison to individuals with suppressed HIV and higher CD4 counts (3) and SARS-CoV-2 viral RNA can persist for months in tissues, including the lymphoid, gastrointestinal, and respiratory tissues (52). In NHP studies, RT-PCR is used to discern input SARS-CoV-2 challenge virus (genomic RNA) from newly replicating virus (subgenomic RNA). In rhesus macaques, WA.1/Wuhan SARS-CoV-2 sgRNA typically clears 7–10 DPI in the BAL and 4–14 DPI from the upper respiratory tract (25, 34, 53–55). In our study, we observed persistent SgRNA in the upper respiratory tract, but not the lower respiratory tract during SIV infection, when compared to historical control data (25, 26). While subgenomic SARS-CoV-2 RNA, may not be a definitive marker of active replication (56), our ability to isolate infectious SARS-CoV-2 virus, which is rarely reported in NHP studies, from multiple samples at 14 DPI provides evidence for persistent infection in our model (2). In contrast to our findings, persistent viral replication was not observed in a similar study of SIV-infected pigtail macaques by Melton et al. (51), potentially due to a smaller sample size or because of differences in macaque species, warranting further investigation into the impact of HIV co-infection on SARS-CoV-2 persistence in NHP models. Consistent with our findings, Hasenkrug et al. recently demonstrated that T-cell depletion in rhesus macaques delayed SARS-CoV-2 viral clearance (54). Collectively these studies demonstrate that while T-cells contribute to SARS-CoV-2 viral control, they are not strictly required for SARS-CoV-2 viral clearance and that SIV infection can alter the timeframe of SARS-CoV-2 viral clearance.

Seroconversion rates and T-cell responses following COVID-19 vaccination can be highly variable in PLWH and are typically lower in individuals with low CD4 counts (<200 cell/µL) or who are not virally suppressed (57, 58). Consequently, these individuals are at a higher risk of breakthrough infections (59, 60), and may require additional booster immunizations to achieve protective immunity (8–10, 61). Our study found that SIV infection hindered the generation of robust T-cell, IgG binding Ab, and neutralizing Ab against SARS-CoV-2 by 7–14 DPI – when anti-SARS-CoV-2 antibodies and T-cells typically emerge in healthy animals (34–36). Interestingly, most animals in our study developed anti-Spike IgM bAbs, but failed to class switch to produce IgG bAbs, warranting further investigation into the contributing mechanisms. Similarly, Melton et al. did not detect SARS-CoV-2 specific T-cells in the periphery or lung, nor the development of anti-Spike IgG or IgA, or nAb against SARS-CoV-2 by 21 DPI (51). In a separate study, CD4 depletion in rhesus macaques delayed or reduced anti-SARS-CoV-2 IgM and IgG responses, although a strong anamnestic recall response was observed upon reinfection (54). Collectively, these studies suggest that CD4 T-cells are critical for the generation of protective immunity against SARS-CoV-2. In addition, other factors in immunosuppressed SIV-infected macaques appear to hamper the generation of an effective anti-SARS-CoV-2 immune responses.

Both HIV and SARS-CoV-2 are known to impair gut barrier function, promote microbial translocation, and alter the host micobiome (47, 49). Two prior studies in NHP have specifically examined the gut microbiome during SARS-CoV-2 and found no significant changes in alpha diversity during the acute phase of infection (48, 62). Significant and sustained gut barrier dysfunction did not occur in our animals, which contrasts with findings from previous studies in NHP (48, 62). Our findings support prior descriptions of the high-level stability of the microbiome during COVID-19 disease, but newly reveal genus-level changes in the gut microbiome. Notably, we observed rapid and sustained depletion of bacteria in the *Streptococcus* genus, which is commonly found in the normal NHP gut microbiome (48). This change was conserved across all animals and observed in analysis of both rectal swab and stool specimens. While many *Streptococcus* species are commensal, some can be highly pathogenic. Due to the limitations of 16S sequencing, it is not possible to resolve the implicated taxa at the species level with available data in this study, leaving it unclear whether pathogenic and/or commensal *Streptococcus* species were reduced during SIV+/SARS-CoV-2+ co-infection. Interestingly, we also detected an enrichment of *Succinivibrio* within the gut of our co-infected animals. While *Succinivibrio* is commonly found in the gut microbiome of wild macaques (63–65), it appears to be less prevalent in research-housed adult macaques (66). Factors such as diet, age, and sex can influence *Succinivibrio* abundance in the gut (63–65), but in our study, SARS-CoV-2 co-infection is likely the primary driver of this change. In humans, *Succinivibrio* is enriched in PLWH or in those exposed to HIV compared to individual without HIV infection or those who have not been exposed (67–69), however in our animal model *Succinivibrio* abundance was low during SIV-infection. Species within the *Succinivibrio* genus play a role in carbohydrate metabolism, particularly in the fermentation of cellulose and carbohydrates to produce succinate and acetate. Succinate is a metabolite associated with inflammation and has been linked to various inflammatory diseases (70) and is also important for intestinal remodeling and maintaining gut integrity (71).

Characterization of the upper airway microbiome in NHP is limited. In rhesus macaques, the primary genera of the nasal microbiome are *Dolosigranulum* and *Corynebacterium* (72). This pattern was also observed in SIV+/SARS-CoV-2+ co-infected macaques in our study. However, unlike healthy rhesus macaques (72), the nasal microbiome of co-infected macaques also shows dominance by the genus *Staphylococcus* and the family Moraxellaceae. Future research is needed to determine whether these observable differences are due to SIV infection or other environmental factors. SARS-CoV-2 infection and the severity of COVID-19 have been variably associated with changes in the respiratory microbiome (42). Our study is the first to examine the impact of SARS-CoV-2 on the NHP airway microbiome. In human studies, the alpha diversity of the nasal and throat microbiome has generally remained unchanged with SARS-CoV-2 infection, though a few studies have reported decreased alpha diversity, most commonly in the oropharyngeal cavity (42). Similarly, microbial diversity in the nasal microbiome was unchanged in our model, but in contrast, alpha diversity of the tracheal/oropharyngeal microbiome increased during early SIV+/SARS-CoV-2+ co-infection. Future studies in NHP and humans are crucial to fully elucidate the long-term effects of HIV and SARS-CoV-2 co-infection on both the respiratory and gastrointestinal microbiome. Specifically, research should focus on how co-infection alters microbial composition, diversity, and metabolism. Additionally, understanding potential interactions across the lung-gut axis will be essential for revealing how these microbial changes may influence local and systemic immune responses, inflammation, and disease progression.

SARS-CoV-2 persistence and intrahost viral evolution are reported in individuals with untreated or advanced HIV (3). Here, we provide evidence of intrahost SARS-CoV-2 evolution in SIV-infected rhesus macaques, which contrasts findings from co-infected pigtail macaques (51). Previous studies have reported changes in the SARS-CoV-2 genome in rectal swabs from rhesus macaques (73); however, we were unable to recover live virus from rectal specimens in our study. SARS-CoV-2 escape from nAb is often linked to mutations in the Spike receptor binding (RBD) or N-terminal (NTD) domains, which can enhance receptor-binding affinity (74). The occurrence of viral mutations arising from immune pressure is rare (74), and given the acute timeframe of our study and the lack of anti-SARS-CoV-2 immunity that is generated, we expect virus mutations to precede immune divergence. While we only observed silent mutations in the Spike surface glycoprotein, we identified 14 nonsynonymous SNPs in other protein coding regions. Notably, two SNPs in ORF1ab of animal T985 were highly enriched by day 14, suggesting successful selection and expansion of this quasispecies.

Four SNPs of interest emerged independently in most macaques. The A215S mutation in the nucleocapsid phosphoprotein, observed in 6/7 animals, is a minor variant that peaked at 1% in global GISAID data in July 2022. Although this is not a defining mutation for any VOC, the A251V mutation was present in Delta lineage viruses and peaked at 9% of global data in July 2021. This suggests that the 251 site may continue to play a role in the evolution of emerging VOCs. Membrane glycoprotein H125Y and ORF6 Q56X have also been reported in humans, peaking at 4% in February 2020 and 1% in September 2021, respectively. The ORF6 Q56X mutation truncates six amino acids from the C-terminus and was strongly selected for in 6/7 animals, reaching a frequency up to 47%. The T10A mutation in ORF6, observed in only seven high quality human-host genomes to date, was found in 5/7 macaques, suggesting potential differential selective pressure between species.

This study has several study design limitations. Outside the scope of this study, the animals had prior SIV infections, so we unfortunately did not have access to samples or data from before the SIV infection. Our study is limited by the lack of comparison to SARS-CoV-2 mono-infected contemporaneous controls. Thus, to increase the robustness of our results, we leveraged publicly available data (25, 26) and historical control specimens (25, 29–32, 37) where applicable, providing additional context for comparing our results with those from SIV or SARS-CoV-2 infected rhesus macaques. Furthermore, as our study exclusively utilized females, not all our reported findings may be directly applicable to males. Additional studies are needed to further validate our findings.

SARS-CoV-2 is typically an acute infection, but about 10% of infected individuals develop symptoms associated with long COVID (PASC) (75), which can affect multiple organ systems, including the gastrointestinal tract, neurological system, heart and lungs. Long COVID symptoms are diverse and it is hypothesized that a persistent SARS-CoV-2 viral reservoir could contribute to this condition through mechanisms such as modulating the host immune response, enhancing inflammation, stimulating cross-reactive autoantibodies or promoting microbial dysbiosis (52). HIV infection similarly dysregulates many of these immune responses and pathways, and PLWH are at a higher risk for PASC (1). The complexity of long COVID makes it difficult to study in humans and acquiring aged nonhuman primates for research poses additional challenges (76). Given that our model exhibits several characteristics that contribute to long COVID, future studies are needed to determine whether the rhesus macaque model of HIV/SARS-CoV-2 co-infection could also serve as an animal model for long COVID. Furthermore, this immunocompromised animal model could be highly valuable for testing new COVID-19 vaccines and therapeutics, particularly those that aim to be suitable and effective in immunosuppressed populations.

## Data Availability

The original contributions presented in the study are publicly available. This data can be found in the NCBI SRA repository using accession numbers SRR33509647-SRR33509831 (PRJNA1260684) and SRR33479424-SRR33479448 (PRJNA1260606). Microbiome data presented in the study are included in the article's supplementary materials and further inquiries can be directed to the corresponding author. The code for these analyses can be found at https://github.com/O-Connor-Lab/SIV-COVID-Pilot.
