# Supplementary material for: SIV/SARS-CoV-2 coinfection in rhesus macaques impacts viral shedding, host immunity, the microbiome, and viral evolution
Source: Front Immunol. 2025 May 20;16:1587688. doi: 10.3389/fimmu.2025.1587688 (PMC12129928; doi:10.3389/fimmu.2025.1587688)
Supplement: Supplementary file 1 [file DataSheet1.docx]

Supplementary Material

# Supplementary Figures

**Supplementary Figure 1. SIV-infected rhesus macaques develop mild SARS-CoV-2 disease.** On days of clinical exams (A) body weight and (B) body temperature were measured and compared to pre-SARS-CoV-2 levels. (A-B) The dotted line indicates no change from pre-SARS-CoV-2 coinfection. In blood, measurements of (C) white blood cells, (D) neutrophils, (E) lymphocytes, (F) monocytes, (G) hematocrit, (H) hemoglobin, (I) alanine aminotransferase, and (J) aspartate aminotransferase. (A-B) Friedman or Kruskal-Wallis Test with Dunn’s post hoc test comparison versus baseline, *p<0.05, ***p<0.001. (C-J) Holm-Sidak's Multiple comparison test versus baseline, *p<0.05.


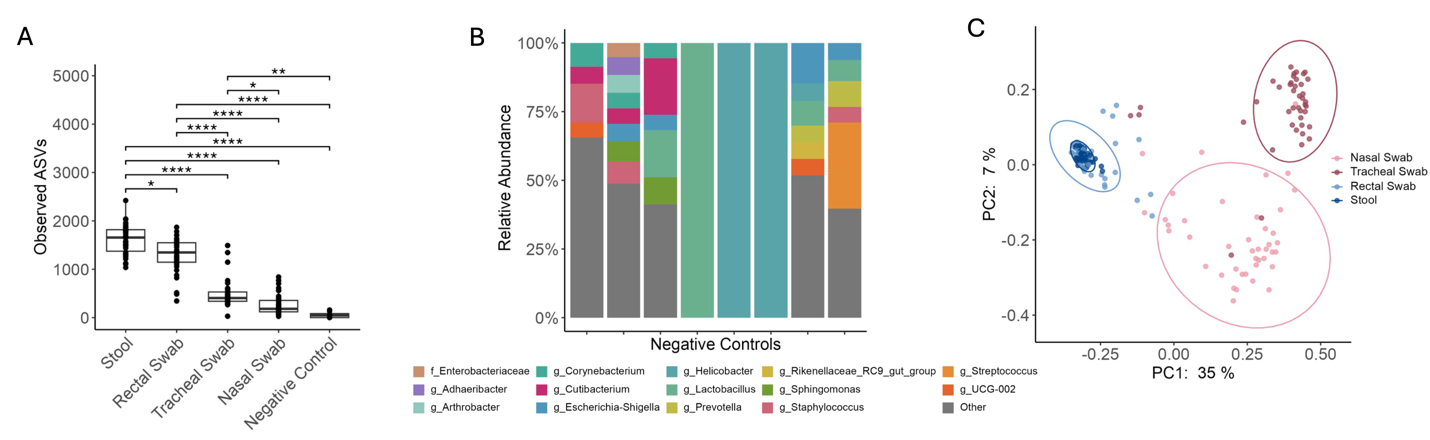


**Supplementary Figure 2. Cross compartment comparisons with controls** (A) Observed features for each location sampled and controls. Alpha diversity was calculated using unrarefied data. Medians with interquartile ranges are shown. The whiskers extend to the largest or smallest value no further than 1.5*IQR from the hinge. Dunn’s Pairwise comparisons with Benjamin Hochberg p-adjustment, * p<0.05, **p<0.01, ****p<0.0001. (B) Relative abundance of the negative controls. Taxa at a relative abundance of less than 5% are included in the Other category. (C) Principal Coordinates Analysis Graph of unweighted UniFrac distance. Ovals represent the 95% confidence interval.

**Supplementary Figure 3. Impact of SARS-CoV-2 on SIV disease progression. (**A) Quantification of SIV viral loads in plasma as determined by RT-PCR. The dotted line indicates the lower limit of detection of the assay (1.79 log_10_ copies/mL). Measurements of blood (B) CD4 counts and (C) %CD4 of total lymphocytes, (D) CD8 counts, (E) %CD8 of total lymphocytes, and (F) CD4/CD8 Ratio. The dotted line indicated a normal CD4/CD8 ratio of 1.0. (A-F) Friedman Test with Dunn’s post hoc test versus baseline, *p<0.05.

**Supplementary Figure 4. Quantification of Sg-E SARS-CoV-2 viral RNA.** Quantification of SARS-CoV-2 subgenomic-E viral RNA in BAL, nasal swab, tracheal swab (n=3) and rectal swab (n=3), as determined by qRT-PCR. The dotted line indicates the lower limit of detection of the assay (50 copies/mL). Friedman Test with Dunn’s post hoc test versus baseline, *p<0.05.

 **Supplementary Figure 5. Evaluation of infectious SARS-CoV-2 post-SIV/SARS-CoV-2 co-infection.** SARS-CoV-2 viral titers per mL of BAL, nasal swab, tracheal swab, and rectal swab, as determined by tissue culture infectious dose assay (TCID50). The dotted line indicates the limit of detection of the assay (2.7 Log_10_ TCID50/mL). Medians are indicated. Wilcoxon matched pairs signed rank test versus baseline, *p<0.05.

**Supplementary Figure 6. SARS-CoV-2 viral persistence in the upper respiratory tract of SIV+/SARS-CoV-2+ rhesus macaques relative to published studies in naïve rhesus macaques.** Comparison of (A) viral and (B) subgenomic-E SARS-CoV-2 RNA at days 7 and 10 in BAL, nasal swab, and/or tracheal swab and rectal swab between SIV+ rhesus macaques (RM) from this study and from historical data of naïve RM from two published studies: (A) Hoang et al., PMCID: PMC7654323 (n=8) and (B) Chandrashekar et al., PMCID: PMC8829873 (n=18). (A-B) Medians are shown. Mann-Whitney test between groups, * p<0.05, ** p<0.01.

**Supplementary Figure 7. Markers of peripheral inflammation and gut integrity.** Quantification of (A) C-reactive protein (CRP), (B) soluble CD14 (sCD14), (C) Intestinal fatty acid binding protein (IFABP), and (D) myeloperoxidase (MPO) in plasma as determined by ELISA. (A) The dotted line indicates the upper limit of detection of the assay (75 μg/mL). (A-D) Friedman Test with Dunn’s post hoc test versus baseline, p-values <0.05 considered significant.


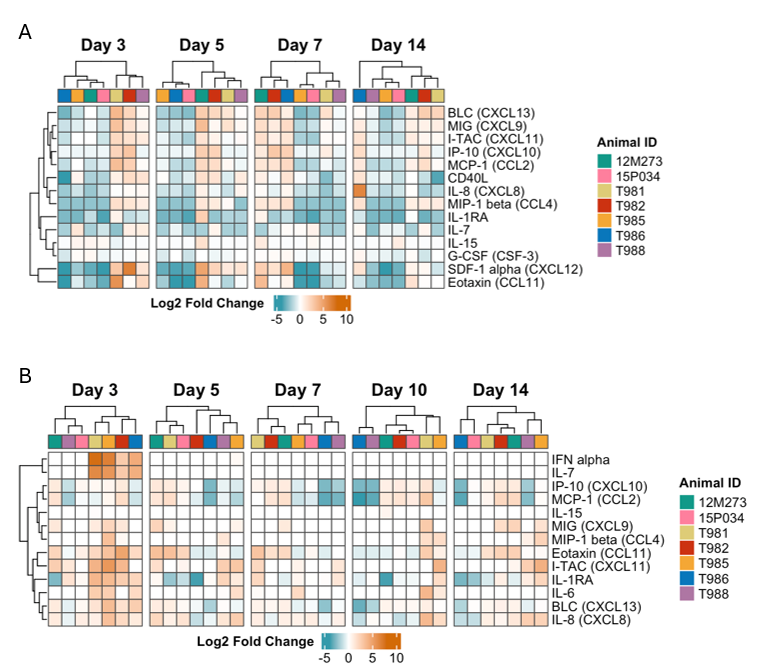


**Supplementary Figure 8. Impact of SIV+/SARS-CoV-2+ co-infection on inflammation.** Concentrations of cytokines and chemokines were determined by multiplex immunoassay in (A) plasma and (B) BAL at the indicated days. Heatmap of Log2 fold-change values from average pre-SARS-CoV-2 baseline levels are shown.

**Supplementary Figure 9. Humoral responses against SARS-CoV-2 VOC.** Serum (A) anti-IgM and (B) anti-IgG enzyme linked immunosorbent assays (ELISAs) against BA.2 and BA.5 Spike proteins. Comparative serum (C) anti-IgM and (D) anti-IgG ELISAs against BA.2 and BA.5 Spike proteins with control specimens (n=19-21). (A-B) Friedman Test with Dunn’s post hoc test versus baseline. (C-D) Medians are shown. Kruskal-Wallis test between groups, * p<0.05, ** p<0.01, ** p<0.001.


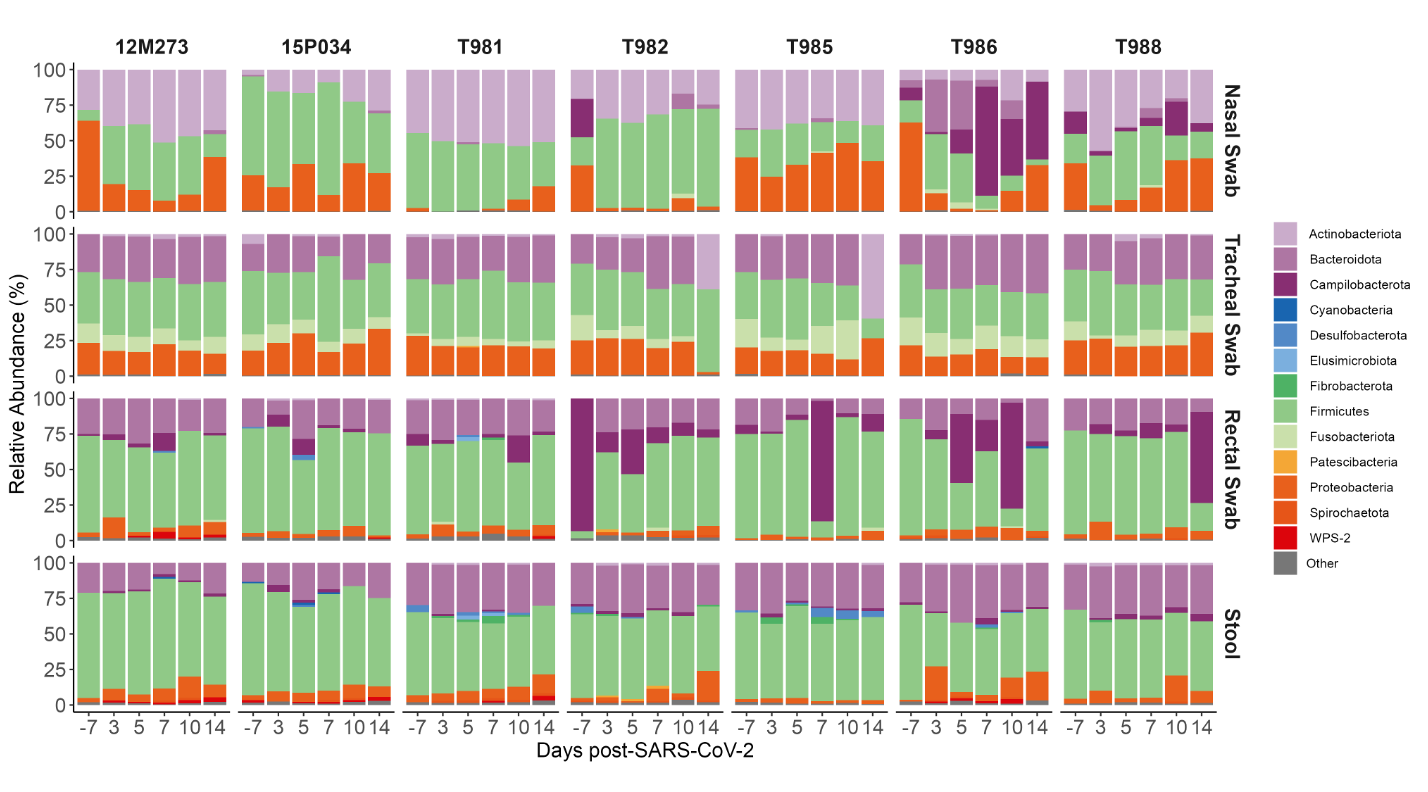
 **Supplementary Figure 10. Relative abundance of the gut and respiratory microbiomes at the phyla level.** Relative abundance of taxa in the respiratory and gastrointestinal microbiomes are classified to the phyla level. Taxa that have an abundance of less than 1% in each sample are pooled into the “Other” category.


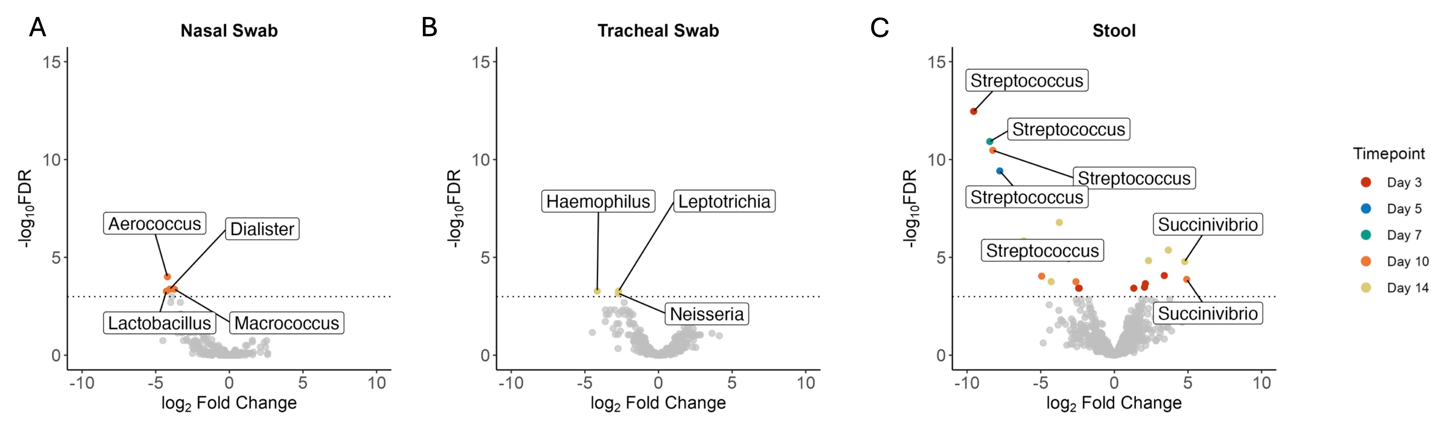


**Supplementary Figure 11. Differential abundance of genera post-SIV+/SARS-CoV-2+ co-infection.** Differentially abundant genera in (A) nasal swabs, (B) throat swabs, and (C) stool as determined by ANCOMBC2. Dotted line indicates a p adjusted value of <0.05. Grey dots represent non-significant taxa, and colored dots represent taxa determined to be significantly abundant on the corresponding day.
